# Contemporary practice about cardiac implantable electronic device infections prevention: A French multicenter survey

**DOI:** 10.1016/j.hroo.2025.10.009

**Published:** 2025-10-23

**Authors:** Antoine Da Costa, Marc Goralski, Aurélie Guiot, Isabelle Lecardonnel, Jean Claude Deharo

**Affiliations:** 1 Department of Cardiology, Jean Monnet University of Saint-Etienne, Saint-Etienne, France; 2 Department of Cardiology, University of Orleans, Orléans, France; 3 Hopital Privé Bois Bernard, Bois Bernard, France; 4 Centre Hospitalier Toulon La Seyne, Toulon, France; 5 Department of Cardiology, AP-HM, La Timone Hospital, Marseille, France

**Keywords:** Cardiac implantable electronic device, Infection, Risk factors, PADIT score, Antibiotic-eluting envelope, Guidelines adherence

## Abstract

**Background:**

Cardiac implantable electronic device infections remain a major complication. Preventive measures include antiseptic skin preparation, systemic antibiotic prophylaxis, and more recently the use of the absorbable TYRX antibiotic-eluting envelope (AEE). Despite explicit international recommendations, real-world adherence to guidelines is insufficiently documented.

**Objective:**

This study aimed to assess infection prevention practices across French hospital centers, with a particular focus on the recent European Society of Cardiology recommendations regarding AEE use.

**Methods:**

Data were collected from 33 French centers (51.3% private, 21.2% academic, 27.3% general hospitals). Each practitioner reported data for 5 patients. A structured questionnaire, validated by 2 international experts, addressed key prevention strategies with specific emphasis on AEE.

**Results:**

Infectious risk assessment was systematically performed, occasionally using risk scores (Prevention of Arrhythmia Device Infection Trial 12.1%; Charlson 3%). Annual infection incidence was most often tracked on a case-by-case basis (54.5%). Screening for *Staphylococcus aureus* carriage was infrequent (18.2%), whereas systematic C-reactive protein measurement was more common (63.6%). Intravenous antibiotic prophylaxis was reported in 93.9% of cases, administered within 1 hour before implantation in 77.4%. Skin preparation with 2% alcoholic chlorhexidine and pocket irrigation with physiological saline were both used in 39.4% of cases. AEE was not systematically applied in reimbursed indications (42.4%), with an overall adherence rate to recommendations of 59.5%.

**Conclusion:**

This multicenter French survey highlights substantial gaps in physicians’ knowledge and application of cardiac implantable electronic device infection prevention practices, despite the availability of clear European Society of Cardiology guidelines.

## Graphical abstract

**Figure:**
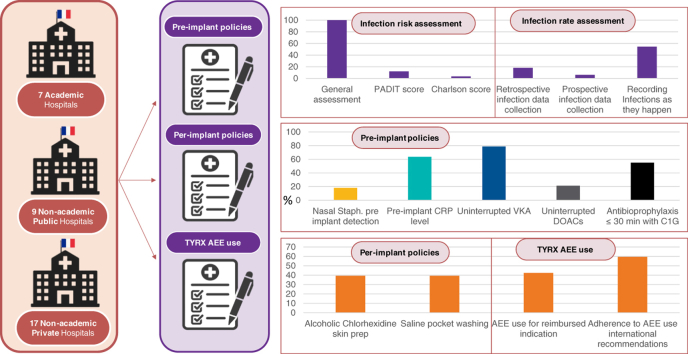

**Key Findings**

▪ Despite multiple practice guidelines and consensus documents on prevention and management of cardiac implantable electronic device (CIED) infections along with multiple attempts to improve their dissemination and implementation, major gaps in knowledge and insufficient adherence to guidelines still remain a challenge.
▪ This French multicenter survey confirmed and emphasized that awareness and follow-up of CIED infection preventive recommendations remain unclear, perhaps not fully implemented in real-world practice.
▪ For example, assessing patient infection risk using the Prevention of Arrhythmia Device Infection Trial score and the antibiotic-eluting envelope use remains largely underutilized in clinical practice.

## Introduction

Cardiac implantable electronic device (CIED) infections remain a major complication of permanent pacemaker and implantable cardioverter-defibrillator (ICD) implantation.^1^, ^2^, ^3^, ^4^, ^5^ The incidence is still rising, attributed to the increasing use of complex devices, reinterventions, and predisposing comorbidities.^3^, ^4^, ^5^ Many known risk factors for CIED infection (CIEDI) are modifiable and thus amenable to preventive strategies aimed at risk reduction.^3^ These preventive measures help limit the primary infection source such as skin antiseptic preparation, systemic antibiotic prophylaxis, and more recently the TYRX antibiotic-eluting envelope (AEE) use.^3^, ^4^, ^5^, ^6^, ^7^, ^8^, ^9^, ^10^, ^11^, ^12^, ^13^, ^14^ The percentage of CIEDI-related events was close to 2% at 1 year but varies according to the Prevention of Arrhythmia Device Infection Trial (PADIT) score.^15^, ^16^, ^17^, ^18^, ^19^, ^20^ The identification of high-risk patients according to the PADIT score, which uses 5 independent clinical and procedural predictors of CIEDI, represents a major risk evaluation approach in real-world practice.^16^, ^17^, ^18^, ^19^ More recently, the novel BLISTER score included additional risk factors identified through multivariate analysis that affected the CIEDI risk, C-reactive protein (CRP) of >50 mg/L, lead extraction, reintervention within 2 years, and top-quartile procedure duration.^20^ In the CIEDI field, the AEE is certainly the new significant major preventive intervention particularly in high-risk populations.^14^^,^^21^ Despite multiple practice guidelines and consensus documents on prevention and management of CIEDIs along with multiple attempts to improve their dissemination and implementation, major gaps in knowledge and insufficient adherence to guidelines remain a challenge.^3^, ^4^, ^5^

Accordingly, this survey aimed to provide an in-depth exploration of infection prevention practices within French hospital centers, with a particular focus on the European Society of Cardiology (ESC) guidelines that recently advocate the use of AEE in very specific situations.^3^, ^4^, ^5^

## Methods

Data collection was based on a structured method in which health care practitioners reported data in groups of 5 patients for each practitioner (cardiologist specialized in cardiac electrophysiology) from 33 French centers. The institutional review committee has waived the need for approval and/or consent because this is an investigation related to implanters’ practices. There is no information about the patients. This study was conducted in accordance with the Declaration of Helsinki on ethical principles for medical research involving human subjects. A specific questionnaire based on the main infection prevention strategies was elaborated and validated by 2 experts (A.D.C. and J.C.D.). Preventive actions for CIEDIs have been categorized in reference to the European preventive recommendations before CIED procedures.^3^, ^4^, ^5^ The prerequisites of the selected centers were to have a uniform protocol of prevention care before implantation. Based on this prerequisite, 17 centers were excluded. The AEE use was specifically studied regarding its recent validation.^3^ Overall, aggregated statistics were reported from blinded responses for survey 1 (general collected data), for survey 2 (AEE indication), and survey 3 (AEE procedure) (Supplemental Tables 1–3). By understanding how AEE is implemented across various clinical settings, the survey seeks to capture insights into the types of prostheses used, associated risk factors, and the procedural details of AEE application. The survey is composed of 3 questionnaires, designed to be completed on the Qualtrics platform, which capture both the overarching preventive practices of medical teams and the specific use in routine patient care.

## Objectives

This survey aimed to provide an in-depth exploration of infection prevention practices within French hospital centers, with a particular focus on the ESC guidelines that recently advocate the use of AEE.^3^, ^4^, ^5^^,^^9^^,^^14^ The primary objective of this analysis was to summarize the preventive action data from a sample of health care practitioners in France. The secondary objective was to evaluate the AEE indication adherence, practice perception, and use according to the European Heart Rhythm Association international consensus of recommended strategies.^1^, ^2^, ^3^, ^4^, ^5^^,^^9^^,^^14^

### Statistical analysis

Descriptive statistics were used to summarize the data collected in the aggregated reports. This includes mean, standard deviation, median, minimum, and maximum for continuous variables and counts and percentages for categorical variables. The sum of the percentages for categorical variables will be higher than 100% if more than 1 choice can be selected in the survey. Summary statistics were reported with a maximum of 2 decimals, as appropriate. The R software, version 4.4.1 (R Foundation, Vienna, AT), was used to perform statistical analysis. No missing data imputation analysis was performed. No hard coding was necessary for this analysis. All authors had full access to the data, took responsibility for its integrity, read the manuscript, and provided final approval.

## Results

### Baseline population characteristics

Overall, aggregated statistics were calculated for 33 responses and were collected for survey 1 (general) (Table 1). In survey 2 (AEE use) (Table 2), indication adherence was studied. The practice use of the AEE was analyzed in survey 3 (procedure) (Table 3). In total, there were 530 CIED implantation procedures analyzed from 35 practitioners in 33 centers, considering the survey structure. Overall, the 33 centers included in the study were delineated as follows: private hospitals in 17 of 33 (51.3%), academic medical centers in 7 of 33 (21.2%), and general hospitals in 9 of 33 (27.33%).

**Table 1:** Questionnaire 1 summary: main CIED procedure prevention parameters

Variable evaluation before CIED implantation	Metric	Summary Statistic
Hospital site	General hospital	9/33 (27.3)
	Academic hospital Private hospital	7/33 (21.2) 17/33 (51.5)
CIED’s infectious risk assessment	PADIT score	4/33 (12.1)
CRP assay	Systematically	21/33 (63.6)
CRP threshold knowledge for delaying the implantation	<50 mg/L	8/33 (27.6)
Anticoagulants management	For VKA agents: continued unchanged with INR 2–3	26/33 (78.8)
	DOAC: stop the day before and the day of the implant procedure whatever the CHADS VA score is	19/33 (57.6)
IV antibiotic prophylaxis	Cefazolin	31/33 (93.9)
IV antibiotic prophylaxis before starting the procedure	Within 1 h	24/31 (77.4)
Skin antisepsis	Povidone–iodine–alcohol 10% chlorhexidine gluconate alcohol 2%	20/33 (60.6) 13/33 (39.4)
Temporary pacing	>25%	10/33 (30.3)
Pocket washing	Povidone–iodine–alcohol 10% solution (eg, Betadine) Physiological solution	6/33 (18.2) 13/33 (39.4)

Data are presented as n/N (%).

CIED = cardiac implantable electronic device; CRP = C-reactive protein; DOAC = direct oral anticoagulant; INR = international normalized ratio; IV = intravenous; PADIT = Prevention of Arrhythmia Device Infection Trial; VKA = vitamin K antagonist.

**Table 2:** Questionnaire 2 summary: survey 2 (AEE use indication adherence)

Variable	Metric	Summary statistic
Recommendation adherence^3^	Systematic	14/33 (42.4)
AEE experience since WRAP-IT study release	> 50	12/33 (36.4)
Saline solution use before AEE	Systematic	29/33 (87.9)
AEE use perception	Needs improvement	24/33 (72.7)
Nonrefunded indications, would you need the AEE?	First implant of CRT pacing	16/33 (48.5)
	High-risk PADIT score of ≥7	15/33 (45.5)
Perception of risks associated with AEE use	No	23/33 (69.7)
AEE perception use for CIED replacement	Complication risks for replacement procedures	2/10 (20.0)
	Procedure prolongation	1/10 (10.0)
	Lead dislodgment risk	2/10 (20.0)
	Risk related to the pocket enlargement	4/10 (40.0)
	Lead damage risk during CIED replacement	2/10 (20.0)
AEE compression bandage practices modification	No	33/33 (100.0)

Data are presented as n/N (%).

AEE = antibiotic-eluting envelope; CIED = cardiac implantable electronic device; CRT = cardiac resynchronization therapy; PADIT = Prevention of Arrhythmia Device Infection Trial.

**Table 3:** Questionnaire 3 summary: survey 3 (AEE procedure)

Variable	Metric	Tachycardia	Bradycardia
Main AEE use indication	Primo implantation	74/355 (20.8)	19/95 (19.5)
	CIED replacement	198/355 (55.8)	63/95 (65.8)
	Upgrading PM or ICD	51/355 (14.4)	8/95 (8.7)
	Lead revision	22/355 (6.2)	4/95 (4.2)
Patients’ age population	<60 y	32/355 (8.9)	16/95 (16.6)
	60–70] y	63/355 (17.8)	27/95 (28.4)
	>70 y	260/355 (73.3)	52/95 (55)
Risk factors considered for AEE use	Age	164/355 (46.2)	42/95 (43.9)
	Moderate CKD	66/355 (18.5)	4/95 (4.2)
	Severe CKD	40/355 (11.3)	16/95 (16.8)
	Terminal CKD	21/355 (5.8)	21/95 (22.1)
	Diabetes mellitus	76/355 (21.3)	34/95 (36.1)
	COPD	38/355 (10.6)	2/95 (2.1)
	NYHA ≥2 heart failure	104/355 (29.3)	13/95 (13.2)
	Cancer	24/355 (6.8)	8/95 (8.9)
	Immunosuppressors agents	24/355 (6.7)	10/95 (10.5)
	Anticoagulant agents use (VKA or direct oral anticoagulants)	97/355 (27.4)	13/95 (13.7)
	≥2 replacements/pocket revision	73/355 (20.6)	38/95 (40.3)
	Reintervention <48 h	13/355 (3.8)	25/95 (26.1)
	Previous CIED infections	22/355 (6.1)	28/95 (29.7)
	Fever	11/355 (3)	8/95 (7.9)
	Type of procedure (revision/replacement)	228/355 (64.2)	56/95 (58.7)
	Type of device (CRT)	112/355 (31.5)	23/95 (24.2)
	Temporary pacing	13/355 (3.7)	2/95 (1.6)
How long the AEE will take to extend the procedure	<1 min	194/355 (54.6)	29/95 (30.5)
	1–5 min	143/355 (40.4)	66/95 (69.5)
	5–10 min	12/355 (3.4)	0/95 (0)
	>10 min	6/355 (1.7)	0/95 (0)
How often do you return the AEE for implantation?	Yes	296/355 (83.4)	79/95 (83.2)
	No	59/355 (16.6)	16/95 (16.8)
Difficulties when inserting the AEE	Yes	79/355 (22.3)	39/95 (41.3)
	No	276/355 (77.7)	56/95 (58.7)

Data are presented as n/N (%).

AEE = antibiotic-eluting envelope; CIED = cardiac implantable electronic device; CKD = chronic kidney disease; COPD = chronic obstructive pulmonary disease; CRT = cardiac resynchronization therapy; ICD = implantable cardioverter-defibrillator; NYHA = New York Heart Association; PM = permanent pacemaker; VKA = vitamin K antagonist.

### Main results

The main results are presented in Supplemental Table 1. Several factors were studied to evaluate the CIED approach implantation: patient infectious risk evaluation based on the PADIT score (12.1%), Charlson score (3%), and traditional medical records (100%); annual infection rate evaluation based on retrospective assessment (18.2%), specific prospective registry (6.1%), as they occur (54.5%), and others (21.2%); identification of nasal carriers of *Staphylococcus aureus* (18.2%); systematic CRP level test measurement (63.6%); anticoagulant strategy, such as continuing vitamin K antagonist agents (78.8%) or direct oral anticoagulants (21.2%) and discontinuing dual antiplatelet agents (no data); prevention strategy, such as preprocedure intravenous antibiotic prophylaxis administration (93.9%) within 1 hour (77.4%), surgical skin preparation by alcoholic 2% chlorhexidine (39.4%), and physiological serum local pocket washing (39.4%); and AEE use indication adherence (Supplemental Table 2). Systematic AEE used in reimbursed French indications was found in 42.4% and the adherence of AEE recommendations in 59.5%. Most implanters thought that using the AEE may favor procedure risks (69.7%). The perception was a procedure time prolongation in 10%, risk of lead dislodgment in 20%, and risk of hematoma in 20% (Supplemental Table 2). The implanters’ main concerns were nonrefunded indications such as primo cardiac resynchronization therapy (CRT) pacemaker indications (48.5%) and high-risk PADIT score of ≥7 reimbursement (45.5%).

### AEE procedure

Many practitioners were reluctant to AEE use owing to the risk of procedure extension time and difficulties of AEE insertion (26.2%) (Supplemental Table 3). They used the AEE in most cases for CIED replacements (58%) and upgrading procedures (13.1%) (Table 1). Risk factors associated with the AEE were extensively described and well known by the implanters (Table 1).

## Discussion

### Major findings

During the last decade, new advances and statements have been published addressing CIEDIs on prevention, diagnosis, and management.^1^, ^2^, ^3^, ^4^, ^5^^,^^9^^,^^14^ However, the rate of infection-related events in the contemporary population remains significantly higher than expected, suggesting that major gaps exist in knowledge and/or insufficient guidelines adherence persist.^3^^,^^4^^,^^22^, ^23^, ^24^, ^25^, ^26^, ^27^, ^28^ This French multicenter survey confirmed and emphasized that awareness and follow-up of CIEDI preventive recommendations remain unclear, perhaps not fully implemented in real-world practice.^1^, ^2^, ^3^, ^4^, ^5^^,^^24^, ^25^, ^26^, ^27^, ^28^ For example, the AEE use remains largely underutilized in clinical practice, mainly owing to unfounded reluctance related to unfamiliarity with the recommended envelope implantation technique.^24^

### CIEDI prevention actions in the real world

Prevention of CIEDI involves risk evaluation and strategies to avoid complications before, during, and after the implantation procedure.^2^^,^^4^ Accordingly, the CIEDI incidence at each center should be available on demand, given that this is the best way to know whether prevention is being performed rigorously and in a personalized manner. This survey found that only 12.2% of centers are prospectively monitoring the incidence of CIEDIs. This is the first point that should be improved in the future.^4^ Increasing awareness of risk factors that can be eliminated or minimized through preventive actions at various levels is also one of the most effective ways to prevent infections.^4^ This survey showed that some preventive measures are underestimated or underused, such as the CRP assay, which is one of the best tools to assess the real infectious risk and decide whether to postpone implantation.^4^^,^^20^ This variable was well documented in the BLISTER score with a discriminative effect of CRP of >50 mg/L as an independent predictor of CIEDIs.^20^ Raised CRP at the time of the implantation measured within the previous 24 hours is strongly associated with CIEDIs and should be incorporated into patient risk assessment.^20^ As presented in Table 1, 63.6% of centers monitored the CRP level the day before implantation and the cutoff value precluding CIED implantation is not very well known (27.6%). The strategy concerning the anticoagulant use is better known and applied based on the ESC recommendations.^3^, ^4^, ^5^^,^^29^^,^^30^ Many studies in the literature have emphasized the importance of continuing oral anticoagulants without bridging.^3^, ^4^, ^5^^,^^24^, ^25^, ^26^^,^^29^^,^^30^ Avoiding the risk of hematoma is the key factor in preventing CIEDIs; therefore, the concomitant use of antiplatelet agents and oral anticoagulants should be avoided.^4^ To achieve this, antiplatelet agents—particularly dual therapy and, if possible, P2Y12 inhibitors—are recommended to be discontinued 5–10 days before surgery.^4^ The most striking finding of this registry concerns the knowledge of antibiotic management before CIED implantation.^4^ The main unexpected finding of this registry concerns knowledge of antibiotic management before CIED implantation. The essential principle of systemic antibiotic prophylaxis—supported by our meta-analysis showing a 75% reduction in CIEDIs—is the administration of cefazolin (1–2 g) within 1 hour before incision.^7^ De Oliveira et al^8^ confirmed our meta-analysis results, demonstrating an 81% reduction in the largest prospective, double-blind, randomized, placebo-controlled trial focused on this topic. However, most centers were unaware of the correct timing for antibiotic administration, regardless of the drug used.^13^ Prophylactic systemic antibiotics covering at least *Staphylococcus aureus* are required, most commonly cefazolin (1–2 g) administered within 1 hour before incision.^4^^,^^7^ The routine use of postoperative antibiotics is not supported by recently published data.^16^^,^^17^ Regarding cutaneous antisepsis before implantation, current recommendations favor the use of a chlorhexidine–alcohol solution. However, French practice remains largely divergent, with a predominant use of 10% povidone–iodine–alcohol solution (60.6%).^2^^,^^4^ Evidence on the prevention of local skin infection during device implantation is limited and largely extrapolated from data obtained in other surgical settings.^4^^,^^11^ To date, only a few retrospective studies have evaluated the preventive role of preoperative antiseptic skin cleansing.^11^^,^^12^ Moreover, there is no clear evidence supporting the benefit of preoperative showering or bathing with chlorhexidine compared with other washing products (eg, bar soap) in reducing surgical site infections.^13^

The effectiveness of preoperative skin preparation depends on both the antiseptic agent and the method used. However, no comparative study has yet been published directly evaluating the 2 available alcoholic solutions (chlorhexidine–alcohol vs povidone–iodine–alcohol).^4^^,^^10^ Although povidone–iodine demonstrates excellent local tolerability, its effects on wound healing and overall efficacy remain controversial. Despite this lack of evidence, 57.8% of centers reported using povidone–iodine as the skin antiseptic of choice, whereas chlorhexidine was reported in 42.2% of centers. However, over time, practice has shifted toward greater use of chlorhexidine–alcohol solutions. For example, in the North American PADIT trial, chlorhexidine–alcohol was used for skin antisepsis in 92.9% of cases (n = 19,603 patients).^16^^,^^17^ To the best of our knowledge, no randomized comparative study has yet assessed chlorhexidine–alcohol vs povidone–iodine–alcohol solutions specifically for the prevention of CIEDIs. Darouiche et al^10^ published a randomized study comparing chlorhexidine–alcohol with aqueous povidone–iodine in general surgery, including both abdominal and nonabdominal procedures (thoracic, gynecologic, and urologic). Chlorhexidine–alcohol was significantly more protective than aqueous povidone–iodine against superficial incisional infections (4.2% vs 8.6%; *P* = .008) and deep incisional infections (1% vs 3%; *P* = .05), but not against organ-space infections (4.4% vs 4.5%).^10^ General recommendations in the literature suggest that the antimicrobial properties of alcohol may constitute the primary “active ingredient” of skin antiseptics.^13^ In addition, prolonged procedure duration (skin-to-skin time ≥120 minutes) has been shown to be associated with a higher risk of CIEDI, with an adjusted hazard ratio (HR) of 2.6 in the BLISTER study.^20^ Importantly, this risk factor has been well integrated into clinical practice, with 66.7% of implanters reporting awareness of its impact.

### CIEDI risk based on PADIT and BLISTER scores

In the field of CIEDIs, maintaining an updated understanding of infection risk factors in standard procedures (permanent pacemaker and ICD implantation) remains of clinical interest, particularly with the real-world application of the PADIT score.^16^, ^17^, ^18^, ^19^ Most studies on this topic are now dated.^19^^,^^20^ More recently, the PADIT score was evaluated in a European study, confirming its utility for identifying high-risk patients. Ziacchi et al^21^ reported similar findings, showing that real-world CIEDI rates were 1.2% in low-risk, 1.7% in medium-risk, and 2.5% in high-risk PADIT categories.^16^ The BLISTER score demonstrated superior discriminative performance compared with the PADIT score, both in standard-risk cohorts (n = 2854; event rate 0.8%; area under the curve [AUC] 0.82 vs 0.71; *P* = .001) and in high-risk validation cohorts (n = 1961; event rate 2.0%; AUC 0.77 vs 0.69; *P* = .001), as well as in the overall population (n = 12,198; event rate 1%; AUC 0.80 vs 0.75; *P* = .002).^20^ In addition to the components of the PADIT score, independent predictors of CIEDI included lead extraction (HR 3.3; 95% confidence interval [CI] 1.9–6.1), elevated CRP of ≥50 mg/L (HR 3.0; 95% CI 1.4–6.4; *P* = .005), reintervention within 2 years (HR 10.1; 95% CI 5.6–17.9), and prolonged procedure duration of ≥120 minutes (HR 2.6; 95% CI 1.6–4.1; *P* = .001).^20^ Despite the dissemination of these findings in recent years and their inclusion in ESC recommendations, only 12.1% of centers reported assessing patient infection risk using the PADIT score, and none used the BLISTER score. This is a critical gap, given that risk stratification is essential for identifying high-risk patients and guiding the use of more intensive preventive measures, such as the application of an AEE.^4^^,^^20^ Recently, an expert group of 8 European cardiologists with expertise in CIED therapy conducted an electronic survey among 302 European cardiologists (18 countries) to evaluate current perceptions and practices regarding CIEDI prevention.^25^ Most respondents (94%) assessed infection risk primarily through clinical evaluation (46%) but applied a risk score in 49%, more than in our French study with only 15% reported.^25^ Commonly adopted preventive measures included intravenous antibiotic prophylaxis (93%), limiting the number of implanted leads (60%), and the use of antibacterial envelopes (57%).^25^ Importantly, 76% of participants expressed the need for clearer, more concise guidelines and more sensitive risk scores to improve preventive strategies.^25^

### Antibacterial envelope use

CIED subgroups at higher risk of infection are now well identified and would warrant the systematic use of an AEE.^3^, ^4^, ^5^, ^6^, ^7^, ^8^, ^9^, ^10^, ^11^, ^12^, ^13^, ^14^, ^15^, ^16^, ^17^, ^18^, ^19^, ^20^, ^21^, ^22^, ^23^, ^24^, ^25^, ^26^, ^27^, ^28^, ^29^, ^30^, ^31^, ^32^, ^33^ The efficacy of AEEs containing 8.0 mg rifampin and 5.1 mg minocycline for pacemaker implantation and 11.9 mg rifampin and 7.6 mg minocycline for ICD implantation was demonstrated in the landmark WRAP-IT trial.^14^^,^^30^ The antibiotics are released into the local tissue over a minimum of 7 days. The latest-generation polymer, composed of glycolide, caprolactone, and trimethylene carbonate, is fully absorbed within 9 weeks. An absorbable polyarylate polymer coating serves as the carrier for minocycline and rifampin.^14^ Both rifampin and minocycline exhibit broad-spectrum activity, penetrate biofilms, and are effective against most staphylococcal species, making them ideal candidates for prophylaxis against CIED pocket infections in high-risk patients. In WRAP-IT, major infections at 12 months occurred in 0.7% of patients receiving an AEE compared with 1.2% in the control group, corresponding to a 40% relative risk reduction.^14^ A model assigning AEEs based on the BLISTER score showed superior efficacy and cost-utility compared with a PADIT-based model.^20^ More recently, Ziacchi et al^21^ reported that AEE use was associated with a >60% reduction in CIEDI events across high-, medium-, and low-risk populations, with the protective effect maintained over time. Surprisingly, in our survey, only 42.4% of implanters reported systematically using AEEs when indicated according to ESC and Haute Autorité de Santé recommendations.^4^ Reluctance to adopt AEE use seems to stem from several uncertainties regarding this technology (Table 3). Paradoxically, in very high-risk situations, some practitioners perceived AEE use as potentially increasing infection risk (Table 3). Ravendren et al^24^ reported a survey in the United Kingdom from 117 participants (79% of whom were consultant cardiologists). This survey provides a snapshot of AEE use across device implanters in the United Kingdom. The authors found that the PADIT score does not reflect the key decision-making factors. Multiple variations in patient selection and practical techniques were evidenced, as well as uncertainty surrounding the clinical evidence and cost-effectiveness of the TYRX envelope.^24^, ^25^, ^26^^,^^31^, ^32^, ^33^ Finally, one of the elements that emerged from this survey is the difficulty of putting the AEE inside the pocket, and many operators fear this situation, expecting a prolonged procedure. The use of this product should be facilitated by a rigorous and clear learning phase, especially in the preparation of the envelope. Furthermore, further studies should clarify which high-risk populations would most likely benefit from AEE implantation during CIED surgery.^14^^,^^21^^,^^30^ Indeed, based on the WRAP-IT study design, patients requiring a de novo CRT pacing were not included; accordingly, this indication is not reimbursed in France.^14^ More recently, a pertinent review by Duncker et al^33^ examined the most recent findings on the effectiveness of antibacterial envelopes in preventing CIEDIs and introduced the latest concept of pocket stabilization. Future studies will have to evaluate the strength of those proposals and the benefits of pocket stabilization in the long term.

### Study limitations

This is a descriptive analysis conducted on a selected set of clinical centers across France. Given the retrospective/prospective observational nature of the research and the absence of individual patient-level monitoring, selection bias cannot be excluded. Data inconsistencies within conditional questions could not be addressed owing to the structure of questionnaires 2–3 (data collected in groups of 5 patients per practitioner; n = 35; 33 centers). The same limitation applied to analyses of patient subgroups. Conduction system pacing, particularly left bundle branch area pacing, may modify infection risk in standard indications; therefore, new studies are required in this specific patient subset. Finally, given that this study focused on French centers, it remains uncertain to what extent the practices summarized here can be extrapolated to other countries. Conversely, recent data from Modi et al^32^ reported that the 2011–2018 US National Inpatient Sample database showed the highest incidence of device-related infection (3.9–4.8%) despite significant technological and procedural advances in CIED design and implantation. Consequently, adherence to guideline-based recommendations remains a primary concern.

## Conclusion

This multicenter French survey demonstrated that, despite clear ESC guidelines, major gaps persist in physicians’ knowledge and skills across all stages of CIED care in real-world practice. Moreover, despite strong scientific evidence, the AEE remains markedly underused, largely owing to unfounded reluctance.

## Disclosures

The authors have no conflicts of interest to disclose.
