# Supplementary material for: Contemporary practice about cardiac implantable electronic device infections prevention: A French multicenter survey
Source: Heart Rhythm O2. 2025 Oct 23;7(1):88–94. doi: 10.1016/j.hroo.2025.10.009 (PMC12902228; doi:10.1016/j.hroo.2025.10.009)
Supplement: Supplementary Tables 1-3 [file mmc1.docx]

# Table 1- Questionnaire 1 – CIED procedure prevention parameters

| **Variable** | **Metric** | **Summary Statistic** |
| --- | --- | --- |
| Hospital site | General Hospital (n/N, %) | 9/33 (27.3%) |
|  | Academic Hospital(n/N, %) | 7/33 (21.2%) |
|  | Private Hospital (n/N, %) | 17/33 (51.5%) |
| How do you evaluate the patient infectious risk ? | Patient records files (n/N, %) | 33/33 (100%) |
|  | PADIT score (n/N, %) | 4/33 (12.1%) |
|  | Charlson score (n/N, %) | 1/33 (3%) |
|  | Others (n/N, %) | 0/33 (0%) |
| In your centre, how do you assess the incidence of CIED infections and decide what preventive measures to put in place? | On request, based on a retrospective review of the coded acts within the department (n/N, %) | 6/33 (18.2%) |
|  | On request, based on the periodic review of infections reported prospectively (registry/filing in the service) (n/N, %) | 2/33 (6.1%) |
|  | As they occur (n/N, %) | 18/33 (54.5%) |
|  | Systematically, based on the periodic review of infections reported prospectively (registry/filing in service) (n/N, %) | 2/33 (6.1%) |
|  | Others (n/N, %) | 5/33 (15.2%) |
|  | No data (n/N) | 1/5 |
|  | No statistics produced (n/N) | 1/5 |
| Before implantation, do you carry out a staphylococcus research by nasal sampling? | Never (n/N, %) | 27/33 (81.8%) |
|  | Rarely (n/N, %) | 6/33 (18.2%) |
| Before an implantation, do you perform a CRP (C-reactive protein) assay? | Never (n/N, %) | 4/33 (12.1%) |
|  | Rarely (n/N, %) | 1/33 (3%) |
|  | Often (n/N, %) | 7/33 (21.2%) |
|  | Consistently (n/N, %) | 21/33 (63.6%) |
| If yes, what CRP threshold do you take to delay implantation and how long? | 5 (n/N, %) | 2/33 (6.9%) |
|  | 10 (n/N, %) | 5/33 (17.2%) |
|  | 15 (n/N, %) | 2/33 (6.9%) |
|  | 20 (n/N, %) | 5/33 (17.2%) |
|  | 30 (n/N, %) | 1/33 (3.4%) |
|  | 50 (n/N, %) | 8/33 (27.6%) |
|  | 80 (n/N, %) | 1/33 (3.4%) |
|  | 100 (n/N, %) | 3/33 (10.3%) |
|  | From 30mg/l (n/N, %) | 1/33 (3.4%) |
|  | Bad indicator in isolation (n/N, %) | 1/33 (3.4%) |
| If yes, how long time do you take to delay the intervention when CRP ≥ 50 mg/l ? - Time (min): | 0-3 days(n/N, %) | 14/33 (42.4%) |
|  | 4-10 days (n/N, %) | 4/33 (12.1%) |
|  | >10 days (n/N, %) | 4/33 (12.1%) |
|  | Other(N/N, %) | 5/33 (15.2%) |
| Before an implantation, what strategy do you adopt towards anticoagulants? | Systematic suspension regardless of anticoagulant, without bridging (n/N, %) | 6/33 (18.2%) |
|  | For VKA agents: continued unchanged with INR 2 - 3 (n/N, %) | 26/33 (78.8%) |
|  | For direct oral anticoagulants continue inchanged (n/N, %) | 7/33 (21.2%) |
|  | For direct oral anticoagulants: stop the day before and the day of implant procedure (n/N, %) | 19/33 (57.6%) |
|  | For direct oral anticoagulants: according to the CHA2DS2 VA SCORE (n/N, %) | 9/33 (27.3%) |
|  | You did sometime an heparine bridge (n/N, %) | 4/33 (12.1%) |
|  | You never did an heparine bridge (n/N, %) | 22/33 (66.7%) |
| **Antibiotic prophylaxis** |  |  |
| Before implantation, which antibiotic(s) are part of the therapeutic arsenal of your practice to prevent infections? (you can specify in the next questions the context of their use) | Cefazolin (n/N, %) | 31/33 (93.9%) |
|  | What choices motivated the use of this antibiotic? | |
|  | First intent choice | 31/31 |
|  |  |  |
|  | Allergy (n/N) | 1/31 |
|  | Other (n/N) | 0/31 |
|  | How often do you use Cefazoline? | |
|  |  |  |
|  | All times except contraindication (n/N, %) | 31/31 |
|  | How long before the operation do you use Cefazoline? | |
|  | 15 min (n/N) | 5/31 |
|  | 30 min (n/N) | 17/31 |
|  | 1h (n/N) | 7/31 |
|  | 2h (n/N) | 2/31 |
|  | Vancomycine (n/N, %) | 29/33 (87.9%) |
|  | What choices motivated the use of this antibiotic? | |
|  | First intent choice (n/N) | 1/29 |
|  | Intensive care or resuscitation leaving (n/N) | 4/29 |
|  | Allergy (n/N) | 28/29 |
|  | Other (n/N) | 2/29 |
|  | How often do you use Vancomycin? | |
|  | Mainly (n/N) | 1/29 |
|  | Sometimes(n/N) | 27/29 |
|  | Often (n/N) | 1/29 |
|  | How long before the operation do you use ? | |
|  | 15 min (n/N) | 5/29 |
|  | 30 min (n/N) | 5/29 |
|  | 1h (n/N) | 15/29 |
|  | 2h (n/N) | 3/29 |
|  | 3h (n/N) | 1/29 |
|  | Clindamycine (n/N, %) | 3/33 (9.1%) |
|  | What choices motivated the use of this antibiotic? | |
|  | Allergy (n/N) | 3/3 |
|  | How often do you use Clindamycine ? | |
|  | Sometimes (n/N) | 2/3 |
|  | Often (n/N) | 1/3 |
|  | How long before the operation do you use Clindamycine ? | |
|  | 30 min (n/N) | 1/3 |
|  | 1h (n/N) | 1/3 |
|  | 2h (n/N) | 1/3 |
|  | Others (n/N, %) | 4/33 (12.1%) |
| For cutaneous antisepsis before implantation, in the absence of allergy, which antiseptic do you use? | Povidone-iodine-alcohol 10% (n/N, %) | 20/33 (60.6%) |
|  | chlorhexidine gluconate 2% and isopropyl alcohol 70% (n/N, %) | 13/33 (39.4%) |
| How long do you wait, usually if you can, before implanting a patient with active infection signs | 72 hours(n/N, %) | 6/33 (18.2%) |
|  | 1 week (n/N, %) | 3/33 (9.1%) |
|  | 2 weeks(n/N, %) | 1/33 (3%) |
|  | Until the infection has cured (n/N, %) | 21/33 (63.6%) |
|  | Based on objective data detailed below: (n/N, %) | 14/33 (42.4%) |
| Generally, in which percentage of cases do you have patients who had temporary stimulation? | <1% (n/N, %) | 6/33 (18.2%) |
|  | [2 – 5] % (n/N, %) | 10/33 (30.3%) |
|  | [6 – 9] % (n/N, %) | 4/33 (12.1%) |
|  | [10 – 25] % (n/N, %) | 3/33 (9.1%) |
|  | > 25 % (n/N, %) | 10/33 (30.3%) |
| What were the indications/profile of these patients who were implanted after their temporary stimulation: | Dependent patient awaiting an implantation (n/N, %) | 29/33 (87.9%) |
|  | United care patients (n/N, %) | 13/33 (39.4%) |
|  | Post-TAVR (n/N, %) | 17/33 (51.5%) |
|  | Material extraction (n/N, %) | 9/33 (27.3%) |
| Do you use and if so what method do you use to wash the pocket? | Never (n/N, %) | 12/33 (36.4%) |
|  | Povidone-iodine-alcohol 10% solution (ex : Bétadine®) (n/N, %) | 6/33 (18.2%) |
|  | Physiological solution (n/N, %) | 13/33 (39.4%) |
|  | Antibiotic solution (n/N, %) | 1/33 (3.0%) |
|  | Other (n/N, %) | 1/33 (3.0%) |
|  | No washing if AEE is used (n/N) | 1/1 |
| How much time do you estimate in your practice the procedure duration according the prothesis type (min)?  Based on the last implantation measurements in each center | | |
| Single chamber pacemaker (PM) : | N | 33 |
|  | Mean ± SD | 27.1 ± 8.7 |
|  | Median (IQR) | 30 (20–30) |
|  | Min–Max | 15–45 |
| Dual chamber PM : | N | 33 |
|  | Mean ± SD | 38.4 ± 12.8 |
|  | Median (IQR) | 40 (30–45) |
|  | Min–Max | 20–70 |
| Cardiac resynchronization PM | N | 31 |
|  | Mean ± SD | 66.9 ± 22.1 |
|  | Median (IQR) | 70 (47.5–90) |
|  | Min–Max | 35–120 |
| Single chamber defibrillator (ICD): | N | 31 |
|  | Mean ± SD | 28.9 ± 8 |
|  | Median (IQR) | 30 (22.5–32.5) |
|  | Min–Max | 15–45 |
| Dual chamber ICD : | N | 31 |
|  | Mean ± SD | 40.6 ± 12.2 |
|  | Median (IQR) | 40 (30–45) |
|  | Min–Max | 20–70 |
| Cardiac resynchronization ICD? : | N | 31 |
|  | Mean ± SD | 72.7 ± 22.2 |
|  | Median (IQR) | 75 (52.5–90) |
|  | Min–Max | 40–120 |
| What time of implantation duration do you consider there is an excess risk of infection? | | |
| Single chamber PM : | [30 – 60 ] minutes (n/N, %) | 2/33 (6.1%) |
|  | [1 – 2 ] hours (n/N, %) | 19/33 (57.6%) |
|  | > 2 hours (n/N, %) | 12/33 (36.4%) |
| Dual chamber PM : | [30 – 60 ] minutes (n/N, %) | 3/33 (9.1%) |
|  | [1 – 2 ] hours(n/N, %) | 16/33 (48.5%) |
|  | > 2 hours (n/N, %) | 14/33 (42.4%) |
| Cardiac resynchronization PM : | [30 – 60 ] minutes (n/N, %) | 1/33 (3%) |
|  | [1 – 2 ] hours (n/N, %) | 10/33 (30.3%) |
|  | > 2 heures (n/N, %) | 22/33 (66.7%) |
| Single chamber ICD : | [30 – 60 ] minutes (n/N, %) | 3/33 (9.1%) |
|  | [1 – 2 ] heure (n/N, %) | 16/33 (48.5%) |
|  | > 2 hours (n/N, %) | 14/33 (42.4%) |
| Dual chamber IC : | [30 – 60 ] minutes (n/N, %) | 2/33 (6.1%) |
|  | [1 – 2 ] hours (n/N, %) | 18/33 (54.5%) |
|  | > 2 hours (n/N, %) | 13/33 (39.4%) |
| Cardiac resynchronization ICD : | [30 – 60 ] minutes (n/N, %) | 1/33 (3%) |
|  | [1 – 2 ] hours (n/N, %) | 10/33 (30.3%) |
|  | > 2 hours (n/N, %) | 22/33 (66.7%) |

# Table 2- Questionnaire 2 – Antibiotic eluting envelope indication use

| AEE TYRX^TM^ use |  |  |
| --- | --- | --- |
|  |  |  |
| When implanting a CIED do you use and AEE as soon as the patient fits the indication? | Sometimes (n/N, %) | 8/33 (24.2%) |
|  | Often(n/N, %) | 11/33 (33.3%) |
|  | Systematically (n/N, %) | 14/33 (42.4%) |
| For what reason(s), would you not use the AEE for an eligible patient? | Not available (n/N, %) | 14/33 (42.4%) |
|  | Pocket too narrow (n/N, %) | 13/33 (39.4%) |
|  | Wrong slip of the envelope (n/N, %) | 17/33 (51.5%) |
|  | Need to expand the pocket (n/N, %) | 14/33 (42.4%) |
|  | Not convinced of its usefulness (n/N, %) | 2/33 (6.1%) |
|  | Allergy (n/N, %) | 6/33 (18.2%) |
|  | Others (n/N, %) | 6/33 (18.2%) |
| How many AEE have you already implemented since the WRAP-IT study release (13)? | Between 1 and 10 (n/N, %) | 3/33 (9.1%) |
|  | Between 10 eand 25 (n/N, %) | 5/33 (15.2%) |
|  | Between 25 and 50 (n/N, %) | 13/33 (39.4%) |
|  | > 50 (n/N, %) | 12/33 (36.4%) |
| Do you put the AEE in saline solution for a few seconds before implantation? | Never (n/N, %) | 1/33 (3%) |
|  | Sometimes (n/N, %) | 2/33 (6.1%) |
|  | Often (n/N, %) | 1/33 (3%) |
|  | Systematically (n/N, %) | 29/33 (87.9%) |
| How would you describe use of the antibacterial envelope? | To improve (n/N, %) | 24/33 (72.7%) |
|  | Difficult (n/N, %) | 3/33 (9.1%) |
|  | Easy to use (n/N, %) | 6/33 (18.2%) |
| For which non-refunded indication(s) would you need the AEE? | First implant of CRT-Pacing (n/N, %) | 16/33 (48.5%) |
|  | First implant of single PM (n/N, %) | 2/33 (6.1%) |
|  | First implant of dual PM (n/N, %) | 3/33 (9.1%) |
|  | First implant of single ICD (n/N, %) | 3/33 (9.1%) |
|  | First implant of dual ICD (n/N, %) | 6/33 (18.2%) |
|  | First implant of SICD (n/N, %) | 6/33 (18.2%) |
|  | High risk PADIT score ≥ 7 (n/N, %) | 15/33 (45.5%) |
|  | Medium rsik PADIT score: [5-6] (n/N, %) | 7/33 (21.2%) |
|  | Low risk PADIT score(n/N, %) | 3/33 (9.1%) |
| Do you identify risks associated with AEE use ? | No (n/N, %) | 23/33 (69.7%) |
|  | Yes (n/N, %) | 10/33 (30.3%) |
| If so, which ? : | Complication risks for replacements procedures (n/N, %) | 2/10 (20.0%) |
|  | Procedure prolongation (n/N, %) | 1/10 (10.0%) |
|  | Lead dislodgment risk (n/N, %) | 2/10 (20.0%) |
|  | Risk related to the pocket enlargment (n/N, %) | 4/10 (40.0%) |
|  | Lead damage risk during CIED replacement (n/N, %) | 2/10 (20.0%) |
|  | Traumatic lesions during AEE manipulation in the pocket(n/N, %) | 6/10 (60.0%) |
|  | Increase risk of bleeding (n/N) | 3/6 |
|  | Tension of the pocket (n/N) | 1/6 |
|  | Hématoma (n/N) | 2/6 |
| Does the use of AEE modify your compression bandage practices | No (n/N, %) | 33/33 (100.0%) |


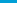

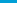


# Table 3 - Questionnaire 3 – AEE Procedure

| Variable | Metric | TACHY | BRADY |
| --- | --- | --- | --- |
| In which procedure type did you use the AEE? | Single chamber PM (n/N, %) | 18/355 (5%) | 20/95 (21.3%) |
|  | Dual chamber PM (n/N, %) | 138/355 (38.9%) | 38/95 (39.5%) |
|  | CRT PM (n/N, %) | 41/355 (11.5%) | 5/95 (5.3%) |
|  | Single chamber ICD (n/N, %) | 20/355 (5.7%) | 4/95 (4.4%) |
|  | Dual chamber ICD (n/N, %) | 40/355 (11.3%) | 6/95 (6.7%) |
|  | CRT-D (n/N, %) | 98/355 (27.5%) | 22/95 (22.9%) |
|  | EV-ICD (n/N, %) | 0/355 (0%) | 0/95 (0%) |
|  | S-ICD (n/N, %) | 0/355 (0%) | 0/95 (0%) |
| What was the main AEE indication? | Primo-implantation (n/N, %) | 74/355 (20.8%) | 19/95 (19.5%) |
|  | CIED replacement (n/N, %) | 198/355 (55.8%) | 63/95 (65.8%) |
|  | Upgrading PM or ICD (n/N, %) | 51/355 (14.4%) | 8/95 (8.7%) |
|  | Lead revision (n/N, %) | 22/355 (6.2%) | 4/95 (4.2%) |
|  | Hematoma pocket revision (n/N, %) | 5/355 (1.4%) | 0/95 (0.5%) |
|  | Controlateral intervention (n/N, %) | 1/355 (0.3%) | 0/95 (0.3%) |
|  | Early revision (n/N, %) | 4/355 (1.1%) | 1/95 (1.1%) |
| What was the age of implanted patients? | <60 years old (n/N, %) | 32/355 (8.9%) | 16/95 (16.6%) |
|  | [60 – 70] years old (n/N, %) | 63/355 (17.8%) | 27/95 (28.4%) |
|  | > 70 yeras old (n/N, %) | 260/355 (73.3%) | 52/95 (55%) |
| What are the infectious risk factors you consider when implanting an AEE? | Age (n/N, %) | 164/355 (46.2%) | 42/95 (43.9%) |
|  | Moderate CKD (n/N, %) | 66/355 (18.5%) | 4/95 (4.2%) |
|  | Severe CKD (n/N, %) | 40/355 (11.3%) | 16/95 (16.8%) |
|  | Terminal CKD (n/N, %) | 21/355 (5.8%) | 21/95 (22.1%) |
|  | Diabete Mellitus(n/N, %) | 76/355 (21.3%) | 34/95 (36.1%) |
|  | COPD (n/N, %) | 38/355 (10.6%) | 2/95 (2.1%) |
|  | NYHA ≥ 2 heart failure (n/N, %) | 104/355 (29.3%) | 13/95 (13.2%) |
|  | Cancer (n/N, %) | 24/355 (6.8%) | 8/95 (8.9%) |
|  | Immunosuppressors agents (n/N, %) | 24/355 (6.7%) | 10/95 (10.5%) |
|  | Anticoagulant agents use (VKA or direct oral anticoagulants (n/N, %) | 97/355 (27.4%) | 13/95 (13.7%) |
|  | ≥ 2 replacements/ pocket revision (n/N, %) | 73/355 (20.6%) | 38/95 (40.3%) |
|  | Reintervention < 48 h (n/N, %) | 13/355 (3.8%) | 25/95 (26.1%) |
|  | Previous CIED infections (n/N, %) | 22/355 (6.1%) | 28/95 (29.7%) |
|  | Fever (n/N, %) | 11/355 (3%) | 8/95 (7.9%) |
|  | Type de procédure (Révision/replacement) (n/N, %) | 228/355 (64.2%) | 56/95 (58.7%) |
|  | Type of device (CRT) (n/N, %) | 112/355 (31.5%) | 23/95 (24.2%) |
|  | Temporary pacing (n/N, %) | 13/355 (3.7%) | 2/95 (1.6%) |
| For patients on anti-coagulants, please specify the following treatments, which are administered: (If you have mentioned a percentage of patients receiving an anti-coagulant, please ensure that the total percentages is 100%) | Heparin (n/N, %) | 12/355 (3.4%) | 1/95 (1.3%) |
|  | VKA (n/N, %) | 74/355 (20.9%) | 8/95 (8.4%) |
|  | Direct oral anticoagulants (n/N, %) | 173/355 (48.8%) | 49/95 (51.8%) |
|  | VKA plus+ antiplatelets agents (n/N, %) | 15/355 (4.2%) | 4/95 (3.7%) |
|  | DOA + antiplatelets agents (n/N, %) | 52/355 (14.6%) | 10/95 (10.8%) |
|  | Antiplatelets agents (n/N, %) | 59/355 (16.7%) | 23/95 (23.9%) |
| How long do you think the AEE will take to extend the procedure | < 1 min (n/N, %) | 194/355 (54.6%) | 29/95 (30.5%) |
|  | [1- 5] min (n/N, %) | 143/355 (40.4%) | 66/95 (69.5%) |
|  | [5- 10] min (n/N, %) | 12/355 (3.4%) | 0/95 (0%) |
|  | Over 10 min (n/N, %) | 6/355 (1.7%) | 0/95 (0%) |
| In case of first-implantation(s) with AEE, what size(s) of pocket do you make compared to a standard pocket size? | Similar (n/N, %) | 88/280 (31.5%) | 28/80 (35.6%) |
|  | Slightly increased (n/N, %) | 168/280 (60.1%) | 52/80 (64.4%) |
|  | Greatly increased (n/N, %) | 24/280 (8.5%) | 0/80 (0%) |
| In case of upgrading/revision/change with AEE use, what extension(s) do you make to the original pocket size? | No modification (n/N, %) | 81/355 (22.7%) | 24/95 (25.5%) |
|  | Slight increase (n/N, %) | 128/355 (36.1%) | 46/95 (48.7%) |
|  | Medium pocket increase (n/N, %) | 115/355 (32.4%) | 25/95 (25.8%) |
|  | Greatly increased (>10% taille de la loge initial) (n/N, %) | 26/355 (7.2%) | 5/95 (5.3%) |
| How often do you return the AEE for implantation? | Yes (n/N, %) | 296/355 (83.4%) | 79/95 (83.2%) |
|  | No (n/N, %) | 59/355 (16.6%) | 16/95 (16.8%) |
| In what sequence do you install the case and AEE? | Insertion the device first in the envelope followed by implantationof both in the pocket (n/N, %) | 340/355 (95.8%) | 93/95 (97.4%) |
|  | Insertion of the TYRX^TM^ first in the pocket followed by the device (n/N, %) | 15/355 (4.2%) | 2/95 (2.6%) |
| Do you insert the leads into the envelope with the device? | Yes (n/N, %) | 126/355 (35.4%) | 32/95 (34.2%) |
|  | No (n/N, %) | 229/355 (64.6%) | 63/95 (65.8%) |
| In case of device replacement, how often was the device brand used different from the previous one? | Different (n/N, %) | 109/355 (30.6%) | 28/95 (30%) |
|  | Identical (n/N, %) | 246/355 (69.4%) | 66/95 (70%) |
| Did you encounter any difficulties when inserting the AEE? | Yes (n/N, %) | 79/355 (22.3%) | 39/95 (41.3%) |
|  | No (n/N, %) | 276/355 (77.7%) | 56/95 (58.7%) |
| In case of difficulties encountered, which device brand(s) of has been used? | Abbott (n/N, %) | 21/225 (9.4%) | 11/85 (12.9%) |
|  | Biotronik (n/N, %) | 34/225 (15.3%) | 14/85 (15.9%) |
|  | Boston (n/N, %) | 32/225 (14.3%) | 10/85 (11.8%) |
|  | Medtronic (n/N, %) | 59/225 (26.3%) | 26/85 (30%) |
|  | Microport (n/N, %) | 43/225 (19%) | 10/85 (11.8%) |
|  | Other (n/N, %) | 35/225 (15.6%) | 15/85 (17.6%) |
